# Glycyrrhetinic Acid Maintains Intestinal Homeostasis via HuR

**DOI:** 10.3389/fphar.2019.00535

**Published:** 2019-05-16

**Authors:** Gang Chen, Bei Bei, Yuan Feng, Xuezheng Li, Zhe Jiang, Jian-Yong Si, De-Gang Qing, Juan Zhang, Ning Li

**Affiliations:** ^1^School of Traditional Chinese Materia Medica, Key Laboratory of Computational Chemistry-Based Natural Antitumor Drug Research & Development, Shenyang Pharmaceutical University, Shenyang, China; ^2^Department of Pharmacy, Yanbian University, Yanji, China; ^3^Institute of Medicinal Plant Development, Chinese Academy of Medical Sciences Peking Union Medical College, Beijing, China; ^4^XinJiang Institute of Chinese Materia Medica and Ethnodrug, Ürümqi, China

**Keywords:** glycyrrhetinic acid, HuR, organoid, fasting, DMFO, licorice

## Abstract

Glycyrrhetinic acid (GA) is one of the main components of the traditional Chinese medicine of licorice, which can coordinate and promote the effects of other medicines in the traditional prescription. We found that GA could promote the proliferation, decrease the apoptotic rate, and attenuate DFMO-elicited growth arrest and delay in restitution after wounding in IEC-6 cells via HuR. GA failed to promote proliferation and to suppress apoptosis after silencing HuR by siRNA in IEC-6 cells. Furthermore, with the model of small intestinal organoids developed from intestinal crypt stem cells, we found that GA could increase HuR and its downstream ki67 levels to promote intestinal organoid development. In the *in vivo* assay, GA was shown to maintain the integrity of the intestinal epithelium under the circumstance of 48 h-fasting in rats via raising HuR and its downstream genes such as EGF, EGFR, and MEK. These results suggested that via HuR modulation, GA could promote intestinal epithelium homeostasis, and therefore contribute to the absorption of constituents from other medicines co-existing in the traditional prescription with licorice in the small intestine. Our results provide a new perspective for understanding the effect of licorice on enhancing the therapeutic effect of traditional prescriptions according to the traditional Chinese medicine theory.

## Introduction

Glycyrrhetinic acid is one of the main components of licorice (*Glycyrrhiza uralensis* Fisch and *Glycyrrhiza inflata* Batal). Having been used in China for around 2,000 years as a traditional Chinese medicine (TCM), nowadays, licorice is also utilized as a resource of food according to the regulations released by the China Food and Drug Administration. Licorice is also known for its high utilization rate, which is commonly described as “nine out of ten prescriptions must have licorice included.” According to traditional Chinese drug theory, licorice has a “coordinating effect” by modulating the effects of other medicines coexisting with it in the same prescription, and thereby improves the therapeutic effect of the prescription, and this special feature of it becomes the main reason of its utilization rate. How could that be happening? So far there is no convincing theory and evidence to explain it. One theory is that licorice might be able to enhance the absorption of molecules from other traditional medicines. As almost all the traditional medicines are used by the way of decoction, it is believed that in the traditional prescription, licorice might be able to maintain and/or restore the intestine homeostasis so as to assure the effective absorption of molecules from other ingredients.

The precise regulation of intestinal epithelium cells (IECs) depends on many factors, such as polyamides, microRNAs, RNA-binding protein, and the stimulation of foods. It is well known that starvation is detrimental to the integrity and the homeostasis of the intestinal mucosa (Mccue et al., 2017), exemplifying the fact that, when feeling sick, we usually have a bad appetite, which lowers our food intake. For those who had a severe disease or were in a coma in ancient times, following a regular diet was not achievable, resulting in the defective intestinal epithelial integrity. Thus, under these conditions, restoring the intestinal integrity and homeostasis are the primary issues to address when using a decoction of traditional medicines that are needed to be absorbed mainly via the small intestine.

Glycyrrhizic acid (a triterpene glycoside with GA as the aglycone) and GA are two well-known components in licorice. As most of the glycosides are hydrolyzed *in vivo* to produce the aglycone, GA is also considered as the active form of glycyrrhizic acid. Thus, in this paper, the *in vitro*, *ex vivo* (organoids), and *in vivo* models were utilized to investigate the effect of GA on intestinal restitution, offering a new perspective on the coordinating effect of licorice.

## Materials and Methods

### Animals and Ethics Statement

SD rats were obtained from the Guangdong experimental animal research center. All experiments were performed in a specific pathogen-free unit of Guangzhou University of Chinese Medicine. The study protocol was approved by the Ethics Committee for Animal Experiments of Guangzhou University of Chinese Medicine (2016-0502).

### Chemicals and Cell Culture

The IEC-6 cell line and Caco-2 cell line were purchased from the Kunming Cell Bank. IEC-6 cells were at passage 15 and were maintained in T-150 flasks in Dulbecco’s modified Eagle’s medium (DMEM) supplemented with 5% heat-inactivated FBS. IEC-6 cells were used at passages 15-20.

High-sugar DMEM cell culture medium was purchased from Hyclone, United States. Fetal bovine serum (FBS), 0.25% Trypsin-EDTA was purchased from BioInd (Biological Industries, Israel). HuR, β-actin antibody was purchased from Cell Signaling Technology, United States. Claudin-1 and Ki-67 antibodies were purchased from Abcam, United Kingdom. GAPDH antibody was purchased from Shenyang Wanlei Biotechnology Co., Ltd. The corresponding secondary antibodies were purchased from Proteintech, United States. PVDF membrane and an immunoblotting chemiluminescence (ECL) system were purchased from Bio-Rad. The reverse transcription kit and Real-time fluorescence quantification kit were purchased from TaKaRa, Japan. siHuR, siSPRY4-IT1, SPRY4-IT1, and Claudin-1 primers were designed and synthesized by Guangzhou Ruibo Biotechnology Co., Ltd. The apoptosis detection kit was purchased from BD Biosciences, United States. The cell cycle detection kit was purchased from Beyotime, Beijing. GA (purity ≥ 98%) was obtained from Phystandard Technology LTD (Tianjin, China).

### Intestinal Organoid Culture

C57BL/6 mice were sacrificed according to applicable ethical regulations and about 20 cm of small intestine were harvested to isolate the intestinal crypts. Crypt fractions were re-suspended in cold DMEM/F-12 and the number of crypts per mL were calculated. The dome containing crypts were incubated and suspended in a 1:1 mixture of Matrigel^^®^^ Matrix (BD Biosciences, United States) and IntestiCult^TM^ organoid growth medium (Stemcell Technologies, Canada) at 37°C and 5% CO_2_, respectively. The culture medium were fully replaced three times per week.

### Flow Cytometry Assay

Annexin V/PI staining assay was employed to determine apoptosis, and apoptosis analysis was carried out using a FITC-Annexin V apoptosis detection kit (BD Biosciences, San Jose, CA, United States) according to the manufacturer’s instructions. Briefly, 1 × 10^6^ cells/well of IEC-6 were cultured in a 6-well plate. The next day, the cells were treated with GA (1, 10, and 20 μM) and incubated for 24 h. At indicated times, cells were harvested, and both attached and floating cells were collected, washed twice with ice-cold PBS, and cells were re-suspended in a 100 μL binding buffer. The next 5 μL of FITC Annexin V and 5 μL PI were added and incubated for 15 min at RT (25°C) in the dark. And 400 μL of binding buffer were added to each tube. The data were collected on a BD FACSVerse flow cytometer (BD Biosciences, San Jose, CA, United States) and were analyzed using FlowJo software. The apoptosis rate was obtained through the following formula:

Apoptosis rate%=(number of apoptotic cells)/(number of total cells observed)×100%

#### Cell Cycle Analysis

Cells were harvested at 24 h post-treatment, fixed with 70% ethanol overnight. The sample was washed with ice-cold PBS and then stained with the cell cycle detection kit (Beyotime, China). The cells were incubated for 30 min at 37°C in the dark. The data were collected on a BD FACSVerse flow cytometer (BD Biosciences, San Jose, CA, United States) and analyzed using FlowJo software. The proliferation index was obtained through the following formula:

Proliferation index%=(S+G2/M)/(G0/G1+S+G2/M)×100%

### Western Blot Analysis

Cells were washed with ice-cold PBS and lysed with lysis buffer (20 mM Tris–Hcl, pH 7.5, 130 mM NaCl, 1% vol/vol TritonX-100, 0.5% wt/vol deoxycholate, 0.1% wt/vol SDS, 1 mM DTT, 10 mM sodium pyrophosphate, 5 mM sodium fluoride, 1 mM phenyl-methylsulfonyl fluoride, and 200 M sodium orthovanadate. L). The protein content was determined by BCA assay. Equal amounts of protein and the Precision Plus Protein Standards (Bio-Rad) were resolved by SDS–PAGE gels (8% gel) and transferred onto PVDF membranes (Bio-Rad, United States). After soaking in blocking buffer (5% non-fat milk), the membrane was incubated overnight with primary antibody HuR (Cell Signaling Technology, United States), followed by horseradish peroxidase-conjugated secondary antibodies. Membranes were exposed to goat anti-rabbit or anti-mouse (Proteintech, United States) secondary anti-bodies. An antibody against β-actin or GAPDH (Cell Signaling Technology, United States) served as an endogenous reference.

### Cell Growth Assay

Cell viability was measured with MTT assays. Briefly, cells were cultured in 96-well plates (2.5 × 10^3^ cells/well) in complete DMEM. After treatment with various concentrations of GA (1, 10, 20, 40, 60, 80, and 100 μM) for 24, 48, and 72 h, the medium was removed and the cells were incubated with 5 mg/mL MTT for 4 h at 37°C and 5% CO_2_. The formazan formed by live cells was dissolved by adding 100 μL of DMSO to each well, and the optical density at 490 nm (OD_490_) was measured to estimate the number of viable cells. The assay was repeated in three-independent experiments.

### Measurement of Epithelial Repair *in vitro*

Cells were plated at 5.0 × 10^4^/cm^2^ in DMEM containing FBS on 60-mm dishes at 37° and 5% CO_2_. The monolayer was wounded by removing part of the monolayer on day 3; Cells were washed 3 times with PBS and serum-free medium were added; repair was assayed 24 and 48 h after wounding. The results were reported as the cell number of wound width covered.

### Caco-2 Barrier Function Assay

The epithelial barrier function *in vitro* was examined by *trans*-epithelial electrical resistance (TEER) using a 24-well transwell plate. The Caco-2 cells were seeded at a density of 2 × 10^4^ cells/well in 24-well transwell insert (pore size 0.4 μm, Corning). After 48 h of culture, the TEER was measured with an epithelial voltmeter (World Precision Instruments, United States), and then the cells were treated with GA (10 μM). 2 h later, cells were treated with LPS (2 μg/mL). The TEER was measured after LPS and GA co-treatment for 2, 4, 6, and 24 h, respectively. The resistance measurements of cell-free filters (blank resistance) were subtracted from that seeded with cells. The resistance value of the filters and fluids was be subtracted in the calculation for each measurement.

### RNA Interference

The expression of HuR was silenced by transfection with specific siRNA. The siHuR was purchased from Ribobio. For each 60-mm cell culture dish, 5 μL of the 50 μM stock duplex siHuR was used. 48 h after transfection using riboFECT^TM^ CP Reagent, cells were harvested for analysis.

### Immunofluorescence Staining

Intestinal organoid smear was fixed with paraformaldehyde, and immunofluorescently stained for HuR and Ki-67 as the protocol. Briefly, the organoid smears were permeabilized with 0.1% Triton X-100 for 20 min at room temperature, and treated with HuR (1:100) and Ki-67 (1:800) antibodies at 4°C overnight. The image was obtained by laser confocal microscope.

### Statistical Analysis

Data are expressed as means ± SEM. Differences between two groups were analyzed using Student’s *t*-test. For the comparison of multiple groups, an analysis of variance (ANOVA) tests were used, followed by the Student-Newman-Keuls test. *P* < 0.05 was considered to be statistically significant.

## Results

### GA Promotes Cell Proliferation and Restitution After Wounding

**FIGURE 1:**
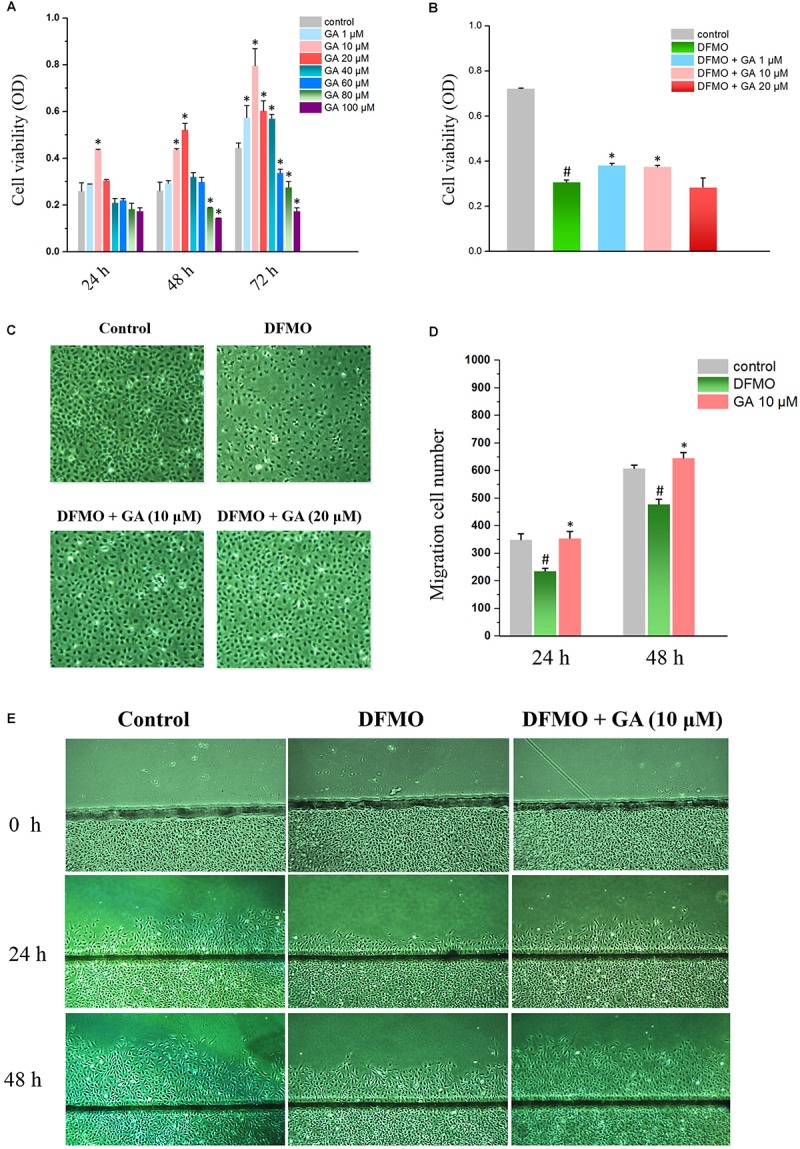
Glycyrrhetinic acid promotes cell proliferation and regeneration after wounding in IEC-6 cells. **(A)** Cell viabilities of IEC-6 cells treated with GA of different concentrations. **(B)** Cell viabilities of IEC-6 cells treated with DFMO and GA of different concentrations. **(C)** Images of IEC-6 cell growth for DFMO and GA groups. **(D)** Summarized data showing rates of migration after wounding for DFMO and GA groups described in panel **(E)**. Values are means ± SEM from six dishes. **(E)** Images of cell migration after wounding for DFMO and GA groups. For panel **(A)** ^∗^*P* < 0.05 compared with control IEC-6 cells. For panel **(D)** ^#^*P* < 0.05 compared with control group and ^∗^*P* < 0.05 compared with DFMO group.

The effect of GA on normal IECs was evaluated in IEC-6 cells by MTT method. The results showed that 10 μM GA promoted cell growth at 24 h. Both 1 and 20 μM GA did not alter the proliferation of IEC-6 cells, while 40–100 μM GA inhibited IEC-6 cell growth after 24 h. As for 48 h GA treatment, both 10 and 20 μM GA enhanced the IEC-6 proliferation, compared with 80 and 100 μM GA that significantly inhibited IEC-6 proliferation. Interestingly, lower doses of GA (1–40 μM) could increase the IEC-6 cell growth, while higher doses of GA (60–100 μM) failed to increase IEC-6 cell growth (Figure 1A). All these results suggested that lower doses of GA, especially 10 μM GA, could promote IEC-6 cell growth. Thus, 1–20 μM GA were adopted to conduct the subsequent experiments.

In order to examine the effect of GA on growth-arrested IEC-6 cells, DFMO was used to deplete the polyamine and thereby inhibit the proliferation of IEC-6 cells. The results showed that DFMO drastically decreased the proliferation of IEC-6 cells. Both 1 and 10 μM GA could partially reverse the growth inhibition caused by DFMO in IEC-6 cells (Figure 1B,C).

Wound healing assay revealed that DFMO suppressed intestinal epithelial restitution, as indicated by a decrease in IEC-6 cell migration, whereas GA could reverse the IEC-6 cell migration arrest elicited by DFMO (Figure 1D,E).

### GA Increases S Phase in IEC-6 Cells

**FIGURE 2:**
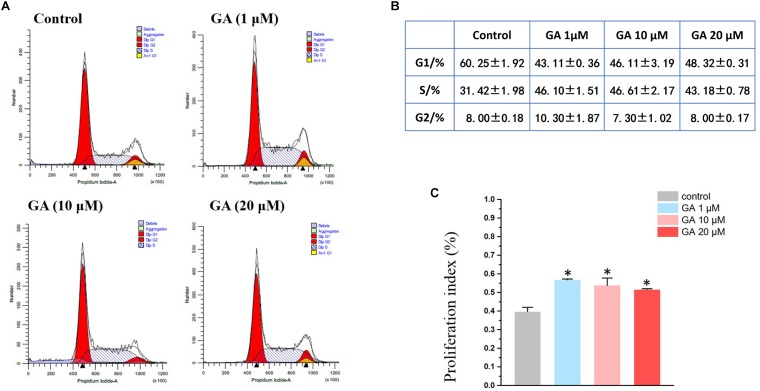
Flow cytometry results show that GA induces S phase arrest in IEC-6 cells treated with GA for 24 h. **(A)** GA induces S phase arrest in IEC-6 cells. **(B)** Detailed cell cycle distribution in IEC-6 cells described in panel **(A)**. **(C)** Summarized data showing proliferation index of IEC-6 cells described in panel **(A)**. Values are means ± SD; *n* = 6 (^∗^*P* < 0.05 compared with the control group).

A flow cytometry experiment showed that GA (1–20 μM) increased the S-phase while decreasing the G1 phase in IEC-6 cells (Figure 2A,B), and led to an increase in cell proliferation as suggested by the index (Figure 2C).

### GA Increases HuR Expression in IEC-6 Cells and Restores the Distribution of HuR in DMFO-Treated IEC-6 Cells

**FIGURE 3:**
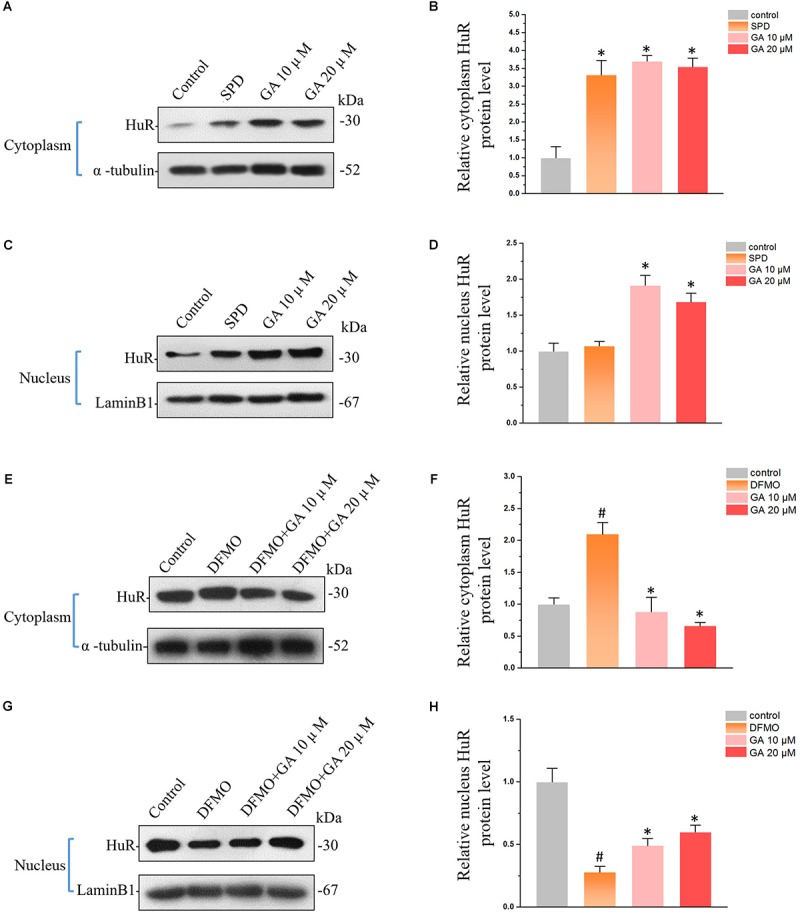
Glycyrrhetinic acid regulates HuR expression in IEC-6 cells. **(A)** Western blot results showing increased HuR levels in cytoplasm by GA and SPD. **(B)** Relative HuR levels in IEC-6 cells described in panel **(A)**. Results are referenced to control group. **(C)** Western blot results showing increased HuR levels in nucleus by GA and SPD. **(D)** Relative HuR levels in IEC-6 cells described in panel **(C)**. **(E)** Western blot results show that GA reversed DFMO-induced HuR overexpression in cytoplasm. **(F)** Relative HuR levels in IEC-6 cells described in panel **(E)**. **(G)** Western blot results show that GA decreases DFMO-induced HuR down-regulation in nucleus. **(H)** Relative HuR levels in IEC-6 cells described in panel **(G)**. For panel **(B,D)**, Values are means ± SEM; *n* = 6. ^∗^*P* < 0.05 compared with the control group. For panel **(B,D)**, Values are means ± SEM; *n* = 6. ^#^*P* < 0.05 compared with the control group and ^∗^*P* < 0.05 compared with the DFMO group.

The HuR protein is the ubiquitously expressed member of the ELAV-like family of RNA-binding proteins (RBPs). Recent studies showed that HuR is one of the key regulators maintaining the intestinal homeostasis post-transcriptionally (Liu et al., 2016). GA increased HuR protein levels both in cytoplasm (Figure 3A,B) and nucleus (Figure 3C,D), exhibiting the same modulatory effect as spermidine (SPD) (Figure 3A–D), one of the polyamines that promotes the proliferation of IECs (Song et al., 2014). The depletion of polyamines by DFMO increased HuR levels in cytoplasm (Figure 3E,F) while decreasing HuR levels in nucleus (Figure 3G,H). It is noteworthy is the fact that GA reversed DFMO-induced aberrant HuR distribution and restored the HuR levels in cytoplasm and nucleus (Figure 3E–H).

### Silencing HuR Eliminates the Effect of GA in IEC-6 Cells

**FIGURE 4:**
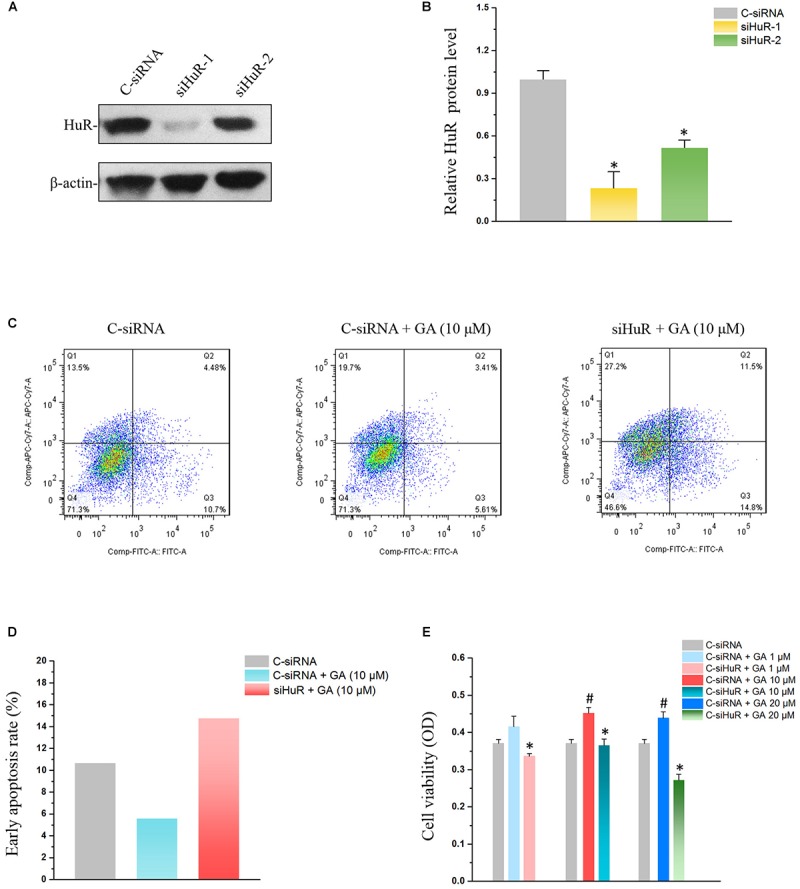
Silencing HuR reverses GA’s effect on IEC-6 cells. **(A)** The siRNA for HuR down-regulates HuR protein levels. Two siRNAs (siRNA1 and siRNA2) were used to down-regulate the HuR protein level, and siRNA1 was adopted in the subsequent experiment for its high efficiency in HuR silence. **(B)** Summarized data of HuR protein levels described in panel **(A)**. Values are means ± SEM; *n* = 6. ^∗^*P* < 0.05 compared with the control siRNA (C-siRNA) group. **(C)** Flow cytometry results showing apoptosis rates of C-siRNA, GA, and GA+siRNA groups. **(D)** Summarized data of apoptosis rates described in panel **(C)**. **(E)** Cell viability results showing the reversing effect of HuR silence on GA-promoted proliferation in IEC-6 cells. Values are means ± SEM; *n* = 5. ^#^*P* < 0.05 compared with the control siRNA (C-siRNA) group and ^∗^*P* < 0.05 compared with C-siRNA + GA groups of various concentration.

In order to examine the relationship between HuR and GA’s effect on IECs, siRNAs were used to silence HuR (Figure 4A,B). Compared with the control siRNA (C-siRNA) group, co-treatment of GA and C-siRNA significantly reduced the early apoptosis rate in IEC-6 cells, while silencing HuR with siRNA totally reversed the GA-induced suppression on IEC-6 cell apoptosis (Figure 4C,D). MTT results showed that GA (1–20 μM) enhanced IEC-6 proliferation at 72 h compared to the C-siRNA group, while GA did not promote IEC-6 cell proliferation after the silence of HuR at 72 h (Figure 4E). These results suggested that GA inhibits apoptosis and promotes proliferation via HuR in IEC-6 cells.

### GA Promotes Rapid Restitution of *Trans*-Epithelial Electrical Resistance (TEER) Impaired by Lipopolysaccharide via Increasing HuR

**FIGURE 5:**
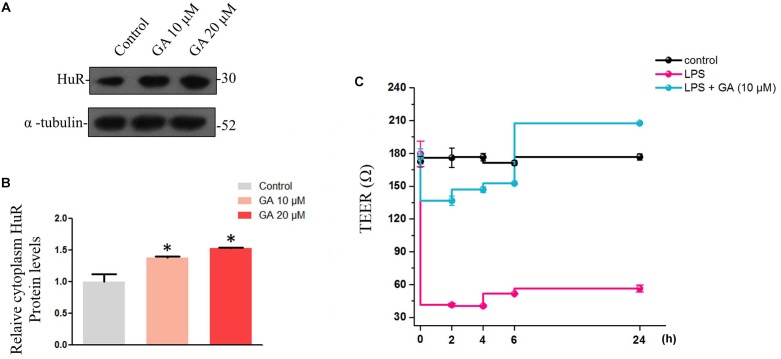
Glycyrrhetinic acid restores the *trans*-epithelial electrical resistance while increasing cytoplasm HuR protein levels in Caco-2 cells. **(A)** Western blot results showing increased cytoplasm HuR levels caused by GA in Caco-2 cells. **(B)** Relative HuR levels in Caco-2 cells described in panel **(A)**. **(C)** TEER values of each group. Values are means ± SEM; *n* = 6. After a 59-h culture, the TEER value of Caco-2 cells becomes stable, and subsequently GA (10 μM) was pretreated 2 h before LPS treatment. ^∗^*P* < 0.05.

The normal intestinal epithelium possesses a barrier function, defending our body from the invasion of endotoxin and of the xenobiotics in the intestine. The maintenance of the intestine barrier function relies on the fast overturn rate of the intestinal epithelium, which would keep the integrity of the intestinal epithelium to maintain its barrier function. Thus, we wondered if GA could promote the formation of the intestinal barrier function by increasing epithelial proliferation. Therefore, Caco-2 cells were used to simulate the intestinal barrier (Haines et al., 2016). Generally, during the Caco-2 cell monolayer formation, the rapid proliferation at the early stage (day 1–3) and the formation of barrier functions, such as tight junction, at later stages (day 3–21) are important. We thus used LPS to interfere with the Caco-2 cell monolayer formation at the early stage and examined if GA rescued the decline in the TEER value caused by LPS through promoting proliferation. In Caco-2 cells, GA increased HuR levels in cytoplasm (Figure 5A,B). For the Caco-2 cell monolayer model, the TEER value became stable at 59 h before reaching the ultimate 600 Ω ⋅ cm^-2^ at later stages. LPS was added at 59 h, and the TEER value of LPS group declined dramatically from 2 h to 24 h after LPS treatment. For the GA group, GA (10 μM) was added 2 h before LPS treatment, and TEER values were measured at 2, 4, 6, and 24 h after LPS treatment. The results showed that GA (10 μM) rescued the decline in TEER value induced by LPS to a relatively normal level in a very short time at the early stage of monolayer formation compared with the LPS group (Figure 5C).

### GA Promotes the Development of 3D Small Intestinal Organoids via HuR

**FIGURE 6:**
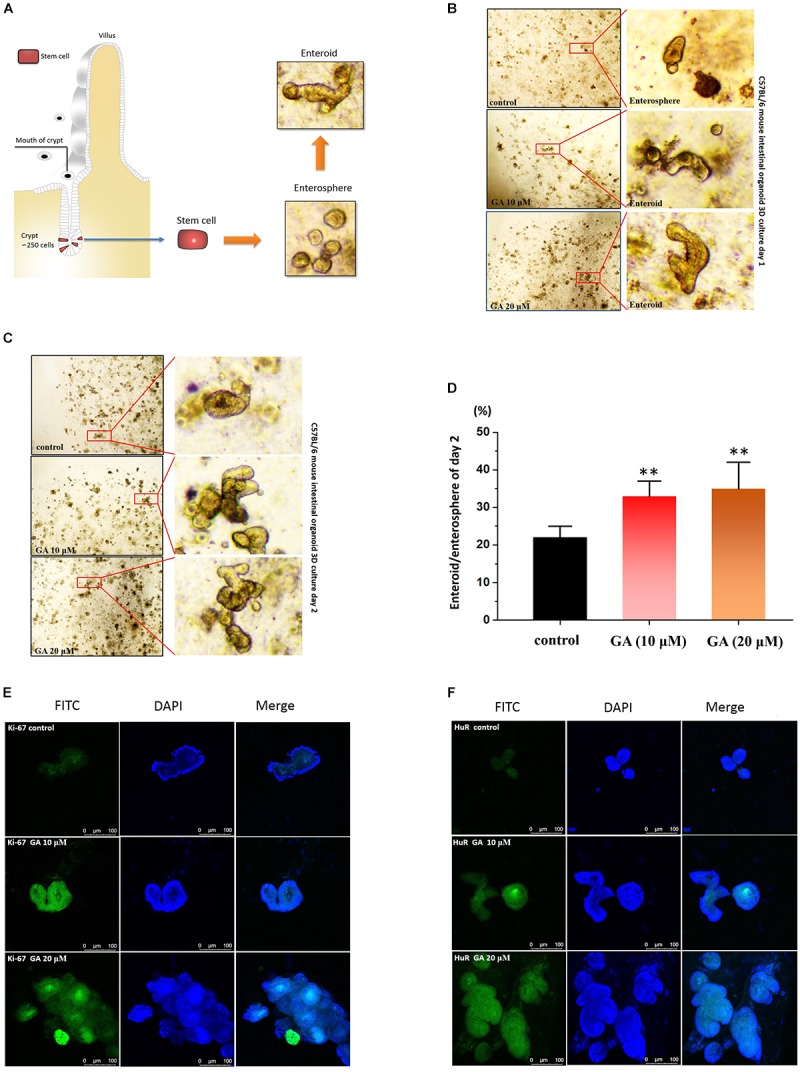
Glycyrrhetinic acid promotes the development of organoids derived from small intestinal crypt. **(A)** The schematic description of small intestinal crypt stem cell isolation and development in to *ex vivo* organoids. **(B)** Development results of day 1 for *ex vivo* culture of C57BL/6 small intestinal crypt stem cells after GA treatment. The isolated stem cells were cultured 24 h before GA treatment. **(C)** Development results of day 2 for *ex vivo* culture of C57BL/6 small intestinal crypt stem cells after GA treatment. **(D)** Ratio of enteroid/enterosphere of organoids derived from small intestinal crypt described in panel **(C)**. Higher ratio of enteroid/enterosphere represents faster development of organoids. **(E)** Immunofluorescence results of ki67 in organoids described in panel **(C)** for each group. **(F)** Immunofluorescence results of HuR in organoids described in panel **(C)** for each group. ^∗∗^*P* < 0.01.

The intestinal epithelium develops through the precise process of “intestinal stem cell differentiation, proliferation and migration, and apoptosis.” Thus, epithelial stem cells derived from C57BL/6 mouse intestinal small bowel were isolated and used to establish *ex vivo* culture of intestinal crypt organoids as a 3D model system for assessing the intestinal epithelium development-promoting effect of GA (Matthias et al., 2012; Figure 6A). GA (10 and 20 μM) significantly accelerated the intestinal organoid development as 24 h after GA treatment, only enterospheres could be found in the control group, while in the GA (10 and 20 μM)-treated groups enteroids were already developed (Figure 6B). Furthermore, the ratio of enteroid/enterosphere was used to evaluate the growth rate of intestinal organoids 48 h after GA treatment (Figure 6C,D). The results showed that GA (10 and 20 μM) potently increased the enteroid/enterosphere ratio, suggesting that GA could drastically stimulate the growth of intestinal organoids. The immunofluorescence results of these *ex vivo* intestinal organoids 48 h after GA treatment showed that the proliferative marker ki-67 was significantly raised by GA (10 and 20 μM) treatment (Figure 6E). Moreover, HuR levels in the *ex vivo* intestinal organoids were also examined, and the results showed that GA (10 and 20 μM) increased the HuR expression while promoting the development of *ex vivo* intestinal organoids (Figure 6F).

### GA Maintains the Small Intestinal Mucosa Integrity via HuR in the Fasting-Induced Intestinal Atrophy

**FIGURE 7:**
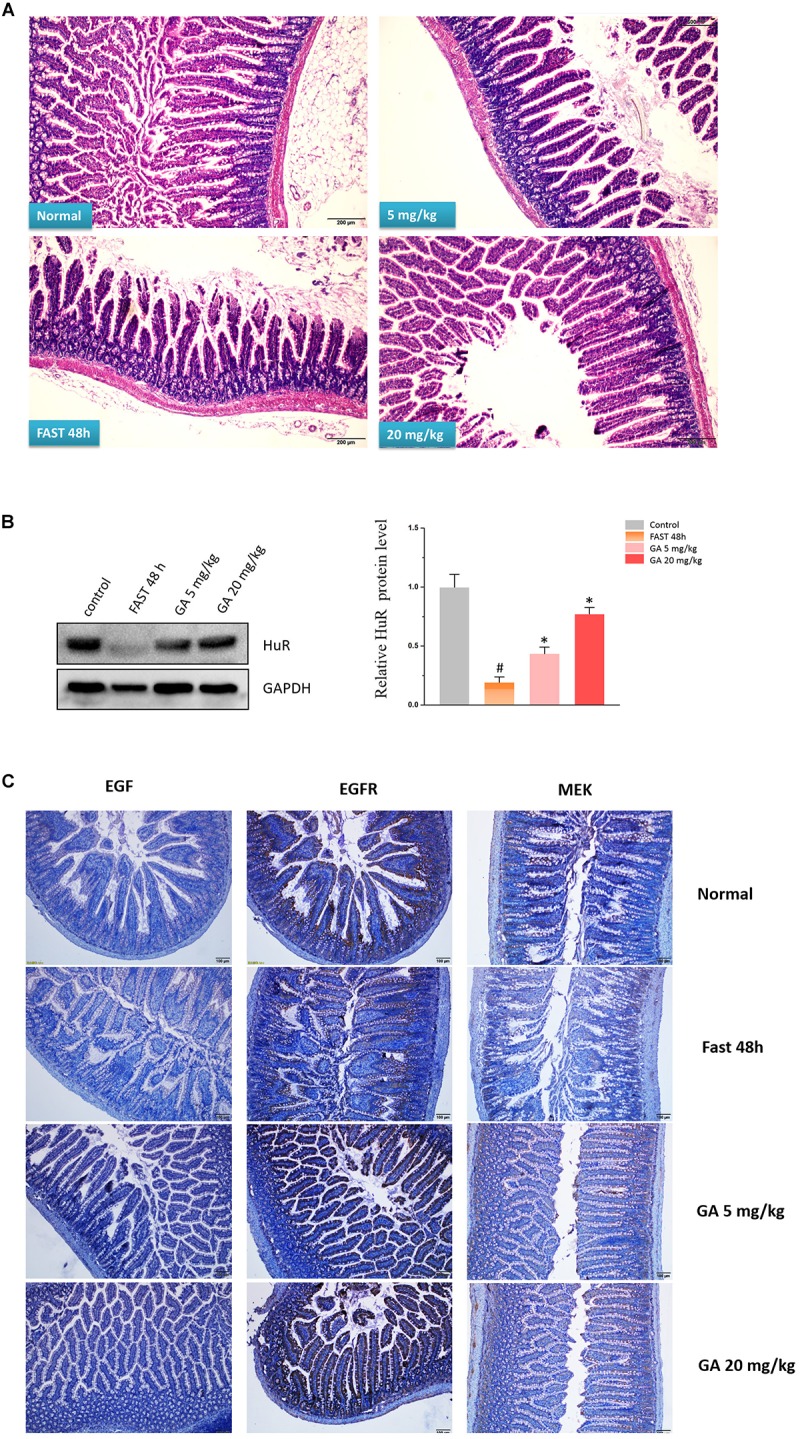
Glycyrrhetinic acid prevents fasting-induced intestinal atrophy through HuR. **(A)** Hematoxylin/eosin staining of the intestinal mucosa. SD rats were deprived of food but allowed free access to tap water for 48 h in the fasting model. Some rats were given GA (5 mg/kg or 20 mg/kg/day once a day) by gavage 24 h before fasting for 3 days (until the end of fasting). Equal amount of normal saline was given in the control group. **(B)** Representative immunoblots of HuR in the small intestinal tissue in rats described in panel **(A)** (left); Relative HuR levels of in the small intestinal tissue in rats described in panel **(A)** (right). ^#^*P* < 0.05 compared with control group and ^∗^*P* < 0.05 compared with FAST group. **(C)** Immunohistochemical results showing EGF, EGFR, and MEK levels in the small intestinal mucosa in rats described in panel **(A)**.

The model of growth inhibition of the small intestinal mucosa by fasting (Uchida et al., 2017) in rats was adopted so as to evaluate the protective effect of GA on the integrity of small intestinal mucosa. In the model group, the integrity of the small intestinal mucosa was severely undermined (being sparse and thin) and was shortened after 48 h of fasting, while both 5 and 20 mg/kg GA administrated 24 h prior to the fasting prevented small intestinal mucosa atrophy (Figure 7A). Western blot results showed that HuR was suppressed by the 48 h-fasting, and GA could restore the down-regulated HuR levels in the model group of fasting (48 h) (Figure 7B). Immunofluorescence results substantiated that levels of EGF, EGFR, and MEK, whose mRNAs have AREs in 3′UTR region and were thereby being regulated post-transcriptionally by RBP HuR (Sun et al., 2016), were all down-regulated by 48 h-fasting, and were also restored by GA administration (Figure 7C).

## Discussion

Licorice has been used as an important TCM. There is a saying that nine of ten TCM prescriptions have licorice included, implying its extensive use in TCM. The reason for its broad adoption in TCM can be partially attributed to the TCM theory, according to which licorice is able to coordinate and promote the effects of other medicines co-existing in the same TCM prescription. The classical explanation on this special effect of licorice is that licorice and its main constituent glycyrrhizin can exhibit mineral-corticoid activity and inhibit atrophy of the adrenal cortex (Sasano et al., 1966a,b), and therefore can usually be used as a remedy in case of emergency. However, licorice is consumed as a food and has been utilized more than just in acute diseases. Thus, another potential mechanism for the “coordination effect” of licorice is maintaining the intestinal homeostasis, especially the small intestinal homeostasis, and thereby promoting the absorption of constituents from TCM in the small intestine.

Previous studies revealed that natural molecules can induce the proliferation of IECs (Nishida et al., 2015), and bioactive components from traditional Chinese medicines can also stimulate intestinal epithelial repair (Song et al., 2017). Among them, licorice and its main components exhibited various functions in the intestine. For example, licorice aqueous extract can regulate polyamine-depleted intestinal crypt cells proliferation (He et al., 2012); GA from licorice can ameliorate intestinal injury elicited by indomethacin or DSS in mice (Ishida et al., 2013; Jeon et al., 2016). However, the effects on IEC proliferation of molecules from licorice, especially GA, remain unknown.

**FIGURE 8:**
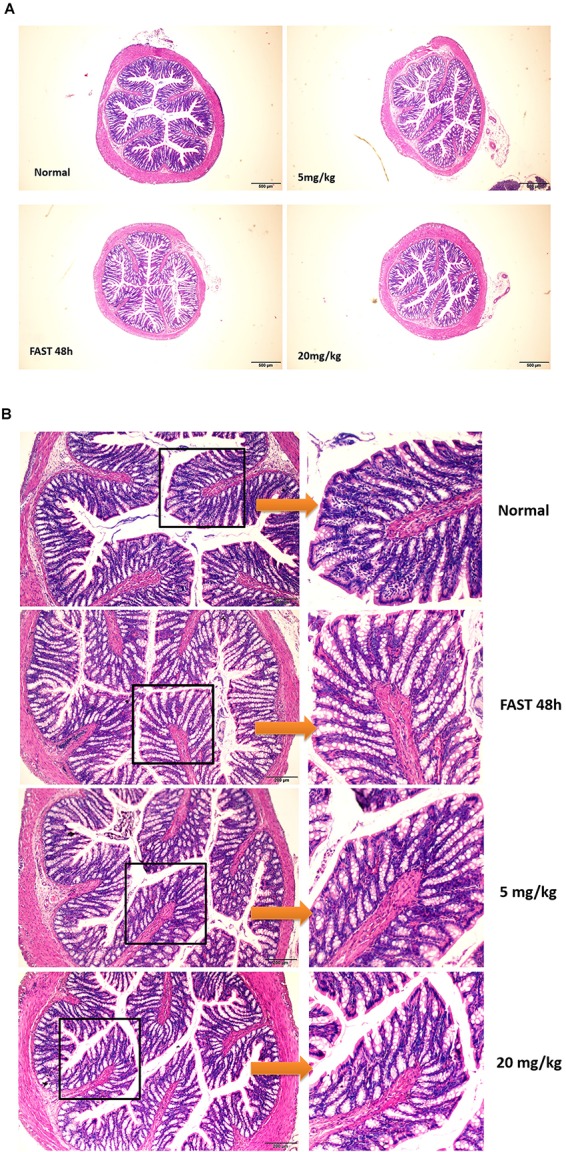
Glycyrrhetinic acid prevents fasting-induced pathological state in colon tissue through HuR. **(A)** Hematoxylin/eosin staining of the colon tissue in SD rats described in Figure 7A. **(B)** Enlarged images of panel **(A)**.

**FIGURE 9:**
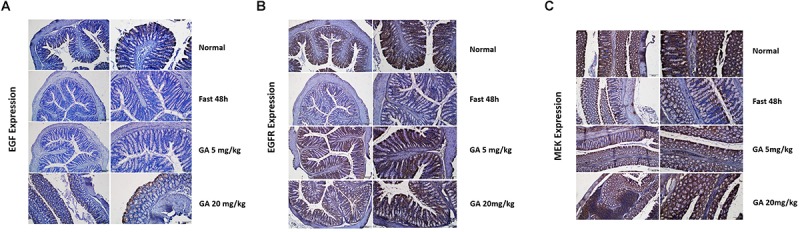
Glycyrrhetinic acid prevents fasting-induced EGF **(A)**, EGFR **(B)**, and MEK **(C)** down-regulation in colon tissue through HuR.

In this paper, we showed that GA could promote the IEC proliferation, facilitate small intestinal organoid development, and prevent fasting-elicited small intestinal mucosal atrophy, indicating a strong protective activity of GA on the small intestine. Furthermore, a similar protective activity of GA was also found in colon tissues. During the 48 h of fasting, the number of caliciform cells and mucus significantly increased in the colon tissue, indicating the initiation of “self-defense” against stress states (Figure 8A,B), while GA administration reduced the aberrant morphological features elicited by fasting to a relative normal state (Figure 8A,B) via up-regulating MEK, EGF, and EGFR (Figure 9), suggesting that besides the protective function in the small intestine, GA could also keep the homeostasis of colon tissue and thereby contribute to the reabsorption of drug molecules to maximize the therapeutic effect of the whole prescription.

**FIGURE 10:**
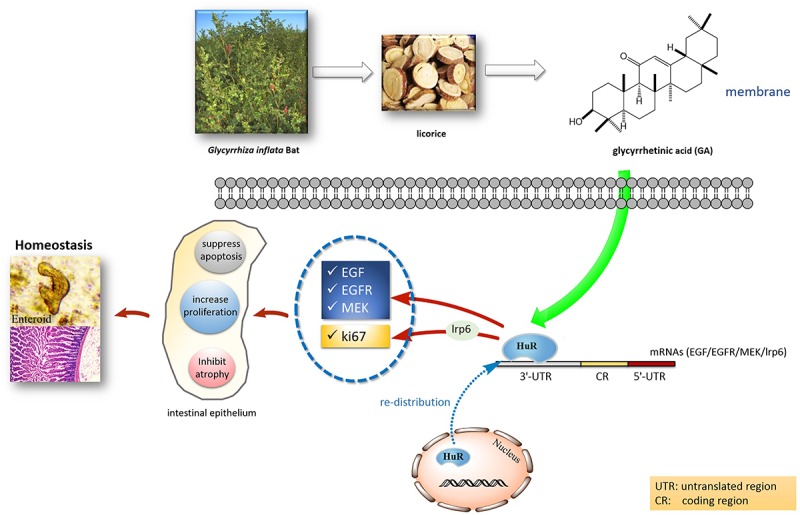
Postulated scheme of GA maintaining intestinal homeostasis via HuR.

Recent studies revealed the important role of HuR in maintaining intestinal homeostasis via the post-transcriptional mechanism (Liu et al., 2016). HuR is a major ARE-binding protein that stabilizes many ARE-mRNAs (Chen et al., 2017). It was suggested that HuR interacts with 3′-UTR of MEK, EGF, and EGFR mRNAs (Sheflin et al., 2004; Sharma et al., 2013). Our previous research showed that HuR can also interact with the mRNA of Wnt co-receptor lrp6 (Li et al., 2016), and thereby could affect proliferative markers such as ki67 in the Wnt signaling pathway. In this paper, we provided evidence that the GA can increase HuR levels and maintain the homeostasis of the intestinal epithelium. Through increasing the HuR expression, GA increases MEK mRNA stability and promotes its translation to elicit the anti-apoptotic response in IECs (Wang et al., 2010; Figure 4C). Moreover, GA was able to increase EGF levels via HuR to attenuate the mucosal atrophy elicited by total parenteral nutrition (TPN) or fasting (Feng et al., 2017), thereby maintaining the intestinal homeostasis. Furthermore, GA-induced HuR overexpression enhanced EGFR and ki67 expression and in turn promoted the proliferation of intestinal epithelial cells (Lee et al., 2015; Sardi et al., 2017) and facilitated the development of small intestinal crypt stem cells (Figure 6B,C). All these data suggest that GA as a main constituent from licorice could maintain intestinal homeostasis under pathological circumstances via modulating HuR, facilitating the absorption of molecules existing in the traditional Chinese decoction (Figure 10). Thus, our results have provided a new perspective for understanding the coordination effect of licorice.

## Ethics Statement

The study protocol was approved by the Ethics Committee for Animal Experiments of Guangzhou University of Chinese Medicine (2016-0502).

## Author Contributions

GC and NL designed the experiments. XL, YF, JZ, and J-YS analyzed the data. GC wrote the manuscript. BB, D-GQ, and ZJ performed the most of the experiments. All authors critically reviewed and approved the final form of the manuscript.

## Conflict of Interest Statement

The authors declare that the research was conducted in the absence of any commercial or financial relationships that could be construed as a potential conflict of interest.

## Abbreviations

ARE: AU-rich elements
DAPI: 4′,6-diamidino-2-phenylindole
DFMO: difluoromethylornithine
EGF: epidermal growth factor
EGFR: epidermal growth factor receptor
FITC: fluorescein isothiocyanate
GA: glycyrrhetinic acid
HuR: Hu-antigen R
LPS: lipopolysaccharide
MEK: MAPK and ERK kinase
MTT: methyl thiazolyl tetrazolium

## References

[B1] Chen J. Adamiak W. Huang G. Atasoy U. Rostami A. Yu S. (2017). Interaction of RNA-binding protein HuR and miR-466i regulates GM-CSF expression. *Sci. Rep.* 7:17233. 10.1038/s41598-017-17371-5 29222492 PMC5722853

[B2] Feng Y. Demehri F. R. Xiao W. Tsai Y. H. Jones J. C. Brindley C. D. (2017). Interdependency of EGF and GLP-2 signaling in attenuating mucosal atrophy in a mouse model of parenteral nutrition. *Cell Mol. Gastroenterol. Hepatol.* 3 447–468. 10.1016/j.jcmgh.2016.12.005 28462383 PMC5403977

[B3] Haines R. J. Jr. Beard R. S. Chen L. Eitnier R. A. Wu M. H. (2016). Interleukin-1β mediates β-Catenin-Driven downregulation of Claudin-3 and barrier dysfunction in Caco2 cells. *Dig. Dis. Sci.* 61 2252–2261. 10.1007/s10620-016-4145-y 27074920 PMC5517031

[B4] He Y. Zhang X. Zeng X. Huang Y. Wei J. A. Han L. (2012). HuR-mediated posttranscriptional regulation of p21 is involved in the effect of *Glycyrrhiza uralensis* licorice aqueous extract on polyamine-depleted intestinal crypt cells proliferation. *J. Nutr. Biochem.* 23 1285–1293. 10.1016/j.jnutbio.2011.07.009 22217517

[B5] Ishida T. Miki I. Tanahashi T. Yagi S. Kondo Y. Inoue J. (2013). Effect of 18β-glycyrrhetinic acid and hydroxypropyl γcyclodextrin complex on indomethacin-induced small intestinal injury in mice. *Eur. J. Pharmacol.* 714 125–131. 10.1016/j.ejphar.2013.06.007 23792039

[B6] Jeon Y. D. Kang S. H. Bang K. S. Chang Y. N. Lee J. H. Jin J. S. (2016). Glycyrrhetic acid ameliorates dextran sulfate sodium-induced ulcerative colitis *in vivo*. *Molecules* 21:523. 10.3390/molecules21040523 27110761 PMC6273862

[B7] Lee M. Kim I. Choi Y. Choi J. Kim Y. Nam T. (2015). The proliferative effects of *Pyropia yezoensis* peptide on IEC-6 cells are mediated through the epidermal growth factor receptor signaling pathway. *Int. J. Mol. Med.* 35 909–914. 10.3892/ijmm.2015.2111 25716690 PMC4356455

[B8] Li Y. Chen G. Wang J. Y. Zou T. Liu L. Xiao L. (2016). Post-transcriptional regulation of Wnt co-receptor LRP6 and RNA-binding protein HuR by miR-29b in intestinal epithelial cells. *Biochem. J.* 473 1641–1649. 10.1042/BCJ20160057 27089893 PMC4888462

[B9] Liu L. Zhuang R. Xiao L. Chung H. K. Luo J. Turner D. J. (2016). HuR enhances early restitution of the intestinal epithelium by increasing Cdc4^2^ translation. *Mol. Cell. Biol.* 37:e574-16. 10.1128/MCB.00574-16 28031329 PMC5359429

[B10] Matthias S. Michael H. Dunn J. C. Y. Henning S. J. Houchen C. W. Calvin K. (2012). A nomenclature for intestinal *in vitro* cultures. *Am. J. Physiol. Gastrointest. Liver Physiol.* 302:G1359-63. 10.1152/ajpgi.00493.2011 22461030 PMC3378093

[B11] Mccue M. D. Passement C. A. Meyerholz D. K. (2017). Maintenance of distal intestinal structure in the face of prolonged fasting: a comparative examination of species from five vertebrate classes. *Anat. Rec.* 300 2208–2219. 10.1002/ar.23691 28941363 PMC5767472

[B12] Nishida M. Murata K. Oshima K. Itoh C. Kitaguchi K. Kanamaru Y. (2015). Pectin from *Prunus domestica* L. induces proliferation of IEC-6 cells through the alteration of cell-surface heparan sulfate on differentiated Caco-2 cells in co-culture. *Glycoconj. J.* 32 153–159. 10.1007/s10719-015-9588-4 25903683

[B13] Sardi C. Luchini P. Emanuelli A. Giannoni A. Martini E. Manara L. M. (2017). Three months of Western diet induces small intestinal mucosa alteration in TLR KO mice. *Microsc. Res. Tech.* 80 563–569. 10.1002/jemt.22831 28094890

[B14] Sasano N. Miyazawa H. Shimizu K. Hirano K. (1966a). Effects of glycyrrhizin on dexamethasone-induced atrophy of the adrenal cortex–histopathological and enzyme histochemical studies. *Nihon Naibunpi Gakkai Zasshi* 42 657–664. 4291268

[B15] Sasano N. Miyazawa H. Shimizu K. Koizumi K. (1966b). Stimulation of rat adrenocortical cells by glycyrrhizin with special reference to its inhibitory effect on dexamethasone-induced atrophy of the adrenal cortex. *Tohoku J. Exp. Med.* 90 391–403. 4291457 10.1620/tjem.90.391

[B16] Sharma S. Verma S. Vasudevan M. Samanta S. Thakur J. K. Kulshreshtha R. (2013). The interplay of HuR and miR-3134 in regulation of AU rich transcriptome. *RNA Biol.* 10 1283–1290. 10.4161/rna.25482 23823647 PMC3817149

[B17] Sheflin L. G. Zou A. P. Spaulding S. W. (2004). Androgens regulate the binding of endogenous HuR to the AU-rich 3′UTRs of HIF-1α and EGF mRNA. *Biochem. Biophys. Res. Commun.* 322 644–651. 10.1016/j.bbrc.2004.07.173 15325278

[B18] Song H. P. Hou X. Q. Li R. Y. Yu R. Li X. Zhou S. N. (2017). Atractylenolide I stimulates intestinal epithelial repair through polyamine-mediated Ca2+ signaling pathway. *Phytomedicine* 28 27–35. 10.1016/j.phymed.2017.03.001 28478810

[B19] Song H. P. Li R. L. Chen X. Wang Y. Y. Cai J. Z. Liu J. (2014). *Atractylodes macrocephala* Koidz promotes intestinal epithelial restitution via the polyamine–voltage-gated K+ channel pathway. *J. Ethnopharmacol.* 152 163–172. 10.1016/j.jep.2013.12.049 24417867

[B20] Sun S. Zhang X. Lyu L. Li X. Yao S. Zhang J. (2016). Autotaxin expression is regulated at the post-transcriptional level by the RNA-binding proteins HuR and AUF1. *J. Biol. Chem.* 291 25823–25836. 10.1074/jbc.M116.756908 27784781 PMC5207058

[B21] Uchida H. Nakajima Y. Ohtake K. Ito J. Morita M. Kamimura A. (2017). Protective effects of oral glutathione on fasting-induced intestinal atrophy through oxidative stress. *World J. Gastroenterol.* 23 6650–6664. 10.3748/wjg.v23.i36.6650 29085210 PMC5643286

[B22] Wang P. Y. Rao J. N. Zou T. Liu L. Xiao L. Yu T. X. (2010). Posttranscriptional regulation of MEK-1 by polyamines through the RNA–binding protein HuR modulating intestinal epithelial apoptosis. *Biochem. J.* 426 293–306. 10.1042/BJ20091459 20001965 PMC3021782

